# Cinnamaldehyde effectively disrupts *Desulfovibrio vulgaris* biofilms: potential implication to mitigate microbiologically influenced corrosion

**DOI:** 10.1128/aem.02200-24

**Published:** 2025-04-28

**Authors:** Arianna Scardino, Gianmarco Mangiaterra, Barbara Citterio, Sarah Hijazi, Caterina Ciacci, Mauro Fehervari, Emanuela Frangipani

**Affiliations:** 1 Department of Biomolecular Sciences, University of Urbino Carlo Bo 541619 , Urbino, Italy; 2 Fano offshore R&D Engineering, Saipem SpA 83398 , Fano, Italy; Washington University in St. Louis, St. Louis, Missouri, USA

**Keywords:** *Desulfovibrio vulgaris*, sulfate reducing bacteria, microbiologically influenced corrosion, biofilm, cinnamaldehyde, eco-friendly alternatives

## Abstract

Microbiologically influenced corrosion poses significant challenges to various industries, as metal surfaces degrade due to the formation of microbial biofilms. Sulfate-reducing bacteria (SRB) are key contributors to this process in anoxic environments (e.g., oil and gas pipelines), mainly by producing highly corrosive hydrogen sulfide. Current prevention methods involving biocides often have drawbacks such as high toxicity and disposal costs, calling for novel environmentally friendly alternatives. Essential oils with their antimicrobial properties and biodegradability may represent promising candidates against microbial corrosion. In this study, cinnamaldehyde was selected both for its antimicrobial activity and its well-documented role as a corrosion inhibitor; then, its antibiofilm activity was investigated against *Desulfovibrio vulgaris*, in comparison with the well-known reference biocide glutaraldehyde. Both compounds were bactericidal against *D. vulgaris* at 12.5 µg/mL. *D. vulgaris* biofilms were developed and monitored by confocal microscopy, and then 72-h-old biofilms were exposed to serial cinnamaldehyde and glutaraldehyde concentrations (12.5–100 µg/mL) for a further 48 h, to evaluate their disruptive effects. Both compounds caused a significant disruption of pre-formed biofilms, at 50 µg/mL. The reduction compared to the untreated controls was ca. 90% vs 85% for biomass, 60% vs 45% for average thickness, and 85% vs 80% for surface area, respectively. Interestingly, cinnamaldehyde applied to a *D. vulgaris* biofilm grown on representative metal coupons completely inhibited the recovery of viable adherent cells. These data, altogether, highlight the potential of cinnamaldehyde as an effective alternative for controlling and mitigating microbiologically influenced corrosion, with comparable efficacy to glutaraldehyde.

**IMPORTANCE:**

The increasing environmental and health concerns associated with the use of conventional biocides to manage and control microbiologically influenced corrosion highlight the need for eco-friendly alternatives. Sulfate-reducing bacteria (SRB) represent the main players in this process, by adhering and proliferating as biofilms on metal infrastructures, producing metabolites that accelerate corrosion. Essential oils have long been regarded as potent antimicrobials endowed with low toxicity; however, there is limited knowledge about their potential use against anaerobic bacteria responsible for corrosion. This study focuses on the antimicrobial activity of cinnamaldehyde and shows its efficacy in eradicating biofilm-grown *D. vulgaris*, a model species to study SRB energy metabolism. Notably, cinnamaldehyde is also a well-known corrosion inhibitor, which makes it an appealing candidate for industrial applications, particularly where SRB-induced corrosion is prevalent. Altogether, our results pave the way for the future development of green sustainable strategies involving the use of cinnamaldehyde to mitigate microbiologically influenced corrosion.

## INTRODUCTION

Microbiologically influenced corrosion is an electrochemical process in which microorganisms can initiate or accelerate metal corrosion reactions (1), enhancing its kinetic rates by 10–1,000 times (2). Indeed, it has been defined by the National Association of Corrosion Engineers (NACE) and the American Society for Testing and Materials (ASTM) as “corrosion affected by the presence or activity, or both, of microorganisms” (3), impacting a wide range of industrial processes, materials and sectors, such as petrochemical installations (4), gas and oil industries (5), water treatment facilities (6), as well as the aviation and defense sectors (7). Microbiologically influenced corrosion causes significant expenses in terms of operational and maintenance costs and may account for up to 20% of the total annual global corrosion damage in the oil and gas sectors, excluding the associated safety and environmental impacts (8). Various microorganisms play a role in this type of corrosion (2), with sulfate-reducing bacteria (SRB) being identified as the primary contributors, especially in anoxic environments (9). SRB contribute to corrosion through multiple mechanisms, the main one being the metabolic production of hydrogen sulfide (H_2_S) during cellular respiration, by reducing the electron acceptor sulfate (10). The presence of H_2_S leads to serious operational problems by reacting with metal ions (mainly iron) and producing ferrous sulfide (FeS), which is poorly soluble in aqueous environments and, together with slime, causes dark-colored sludge that hinders the flow in oil and gas pipelines and also complicates maintenance efforts, contributing to clogging issues during cleaning operations (11). In addition, some SRB species may also generate organic acids, such as acetic and propionic acids, further acidifying the environment and accelerating metal corrosion (12).

Typical SRB include *Desulfovibrio* spp., *Desulfobacter* spp., and *Desulfotomaculum* spp., which are detected on the inner surface of steel rust layers, where the abundance index of SRB is much higher compared to other bacteria species (13). To study SRB energy metabolism and their impact on metal corrosion, the Gram-negative bacterium *Desulfovibrio vulgaris* Hildenborough ATCC 29579 has been widely used as a model microorganism (14). Moreover, *D. vulgaris* has been extensively studied due to its ability to form biofilms (i.e., dense aggregations of microorganisms embedded in a self-produced extracellular matrix). Bacterial biofilms strongly adhere to metallic surfaces, thereby generating and maintaining oxygen-deprived zones that select, promote, and support the growth of specific anaerobic bacteria, such as SRB, thus positively affecting the kinetics of the microbial corrosion process (15). Indeed, biofilms facilitate the local presence of high concentrations of bacteria on metal surfaces, leading to severe degradation, especially on carbon steel materials, which are more vulnerable to SRB biofilms compared to stainless steel ones (16). Moreover, biofilm-growing bacteria exhibit a very high tolerance to external stresses, and their recurrent exposure to biocides used during cleaning and disinfection procedures raises concern about the adaptation routes they might evolve, both at single-cell and community levels (17). When SRB grow as biofilms, they influence the electrochemical conditions of metal surfaces (e.g.*,* carbon steel) by acting as cathodic depolarizers. Indeed, some SRB possess hydrogenases that produce H_2_, which acts as an electron donor for sulfate reduction to H_2_S, thus accelerating the dissolution of the metal (i.e., iron) (18). Moreover, SRB biofilms create localized microenvironments with altered pH and ion concentrations, physically interact with metal surfaces via cell wall components or extracellular polymeric substances (EPS), potentially disrupting protective oxide films and increasing susceptibility to localized corrosion (19–22). To prevent and limit microbiologically influenced corrosion in industrial applications, multiple strategies are currently in use aiming at fighting the adhesion and biofilm formation of microorganisms on metallic surfaces. To these aims, a conventional approach involves the use of biocides (i.e.*,* compounds that kill microorganisms or inhibit their growth), that can be both inorganic compounds (e.g., chlorine, ozone, bromine) and organic ones (e.g., isothiazolones, quaternary ammonium salts, aldehydes such as glutaraldehyde and acrolein) (23–26). Glutaraldehyde is widely used as a biocide, due to its broad-spectrum activity, solubility, and stability across a wide pH and salinity range (27). Moreover, glutaraldehyde is also cost-effective and can be used individually or in combination with other compounds such as quaternary amines and nitrites to reduce the concentration needed to control SRB growth (28). However, glutaraldehyde is toxic to aquatic organisms with long-lasting effects, requiring expensive and complex disposal procedures (29). The environmental apprehensions linked to the documented hazards to human health, together with the reported evidence that glutaraldehyde can be as corrosive to low-carbon steel materials as SRB (30), underscore the need for innovative, effective, and environmentally safe alternatives to replace toxic biocides. This need aligns with the mission area of the Horizon Europe framework program, specifically focusing on the health of oceans, seas, coastal, and inland waters, started in 2021 (31). In this context, the development of bio-inhibitors, such as plant extracts, demonstrated significant potential as effective corrosion inhibitors in various harsh environments (32, 33). Plant extracts serve as a valuable source of naturally occurring chemical compounds that are biodegradable and can be extracted using simple and low-cost methods (34). The effectiveness of these natural compounds in inhibiting corrosion largely depends on the type of metal and their interaction with the surface. Cinnamaldehyde, a naturally bioactive compound, is widely available in the environment, primarily from the bark of cinnamon trees (35). It exhibits antibacterial, antifungal, insecticidal, acaricidal, and nematocidal properties (36–43). Cinnamaldehyde is primarily used in agriculture, food, medical, and flavor and fragrance industries. Its low toxicity, eco-friendliness, and well-known strong adsorption properties make it a promising candidate as an effective green corrosion inhibitor. Moreover, besides its antibacterial activity, cinnamaldehyde has been reported to inhibit acid corrosion (i.e., HCl 10% w/w), due to its ability to form a protective macroscopic film on metal surfaces (33, 44–51). Interestingly, while numerous studies have highlighted the wide antibacterial properties of plant extracts and essential oils (EOs), very few studies have been focused on bacteria involved in microbial corrosion, especially on SRB (52–54). The innate ability of EOs as well as cinnamaldehyde to inhibit corrosion, its minimal toxicity combined with its antibacterial properties, offers a compelling approach to managing the proliferation of SRB in natural settings. In this study, the activity of cinnamaldehyde against biofilm-growing *D. vulgaris* on glass and metal surfaces has been investigated for the first time and compared to the well-known biocide, glutaraldehyde. Cinnamaldehyde was found to be bactericidal at concentrations ranging from 12.5 µg/mL to 100 µg/mL both on planktonic and sessile *D. vulgaris* cultures, with a comparable efficacy to the conventional biocide glutaraldehyde. Moreover, cinnamaldehyde caused a significant disruption of pre-formed *D. vulgaris* biofilms, highlighting its potential as an effective environmentally-friendly alternative for controlling and mitigating microbial corrosion.

## MATERIALS AND METHODS

### Bacterial strain and culture conditions

*Desulfovibrio vulgaris* Hildenborough strain ATCC 29579 (DSM 644) was obtained from the Leibniz Institute DSMZ (Braunschweig, Germany) and grown anaerobically in Hungate tubes filled with Desulfovibrio (Postgate) medium, prepared according to the DSMZ guidelines (Medium N. 63), with minor modifications. Indeed, FeSO_4_ × 7H_2_O concentration was lowered from 0.5 g/L to 0.02 g/L, to limit FeS precipitates formed during *D. vulgaris* growth. Each Hungate tube containing the medium was purged with an N_2_ flux to remove dissolved O_2_ and obtain anoxic conditions, and then sterilized by autoclaving. For solid media preparation, 15 g/L of agar was added prior to autoclaving. *D. vulgaris* was incubated in a standard anoxic environment for 48–72 h at 37°C using a BD GasPak EZ Standard Incubation Container equipped with a BD GasPak EZ Container System Sachet with Indicator to allow the growth of anaerobes. All culture manipulations were performed in the “La Petite” Glove Box (Plas Labs, Inc.), to limit O_2_ concentration. *D. vulgaris* stock solutions were kept as 15% glycerol suspensions at −80°C.

### Determination of MIC and MBC

**Fig 1:**
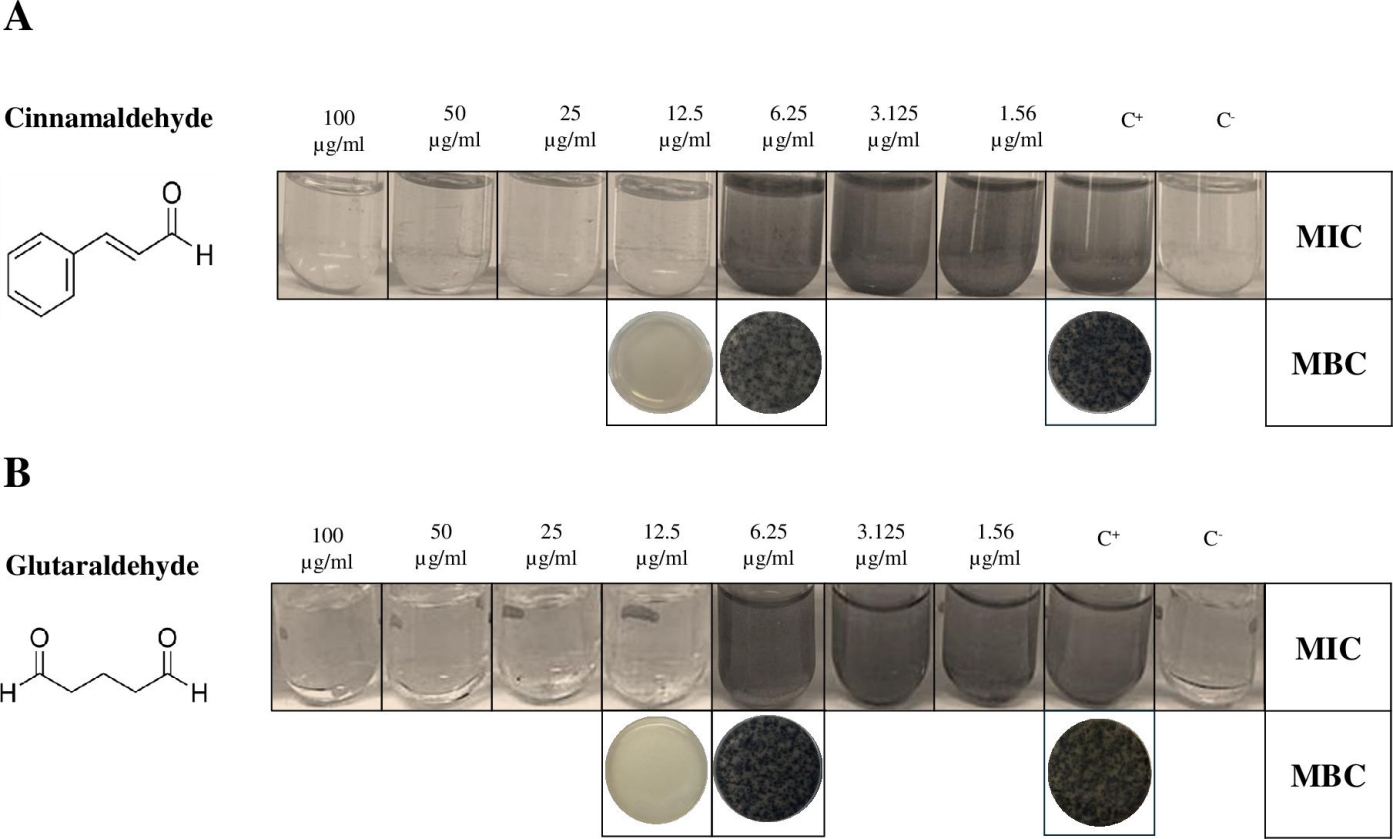
MIC and MBC determination. The MIC of cinnamaldehyde and glutaraldehyde was determined by the broth macrodilution method as the lowest amount of compound that did not result in the rise of turbidity and/or in blackish color of the medium, after 48 h of incubation. The MBC was determined as the first compound dilution for which no visible growth of *D. vulgaris* as colonies on solid media was obtained (representative images from three independent replicates are shown). C^+^ and C^-^ indicate positive and negative controls, respectively. DMSO (i.e., cinnamaldehyde solvent) did not affect growth, at all concentrations tested (data not shown).

Cinnamaldehyde (W228613, Sigma-Aldrich) and glutaraldehyde (G6257, Sigma-Aldrich) were used for susceptibility testing. The minimum inhibitory concentration (MIC) of the selected compounds was determined by following the broth macrodilution method, according to the Clinical and Laboratory Standards Institute guidelines (55). Briefly, 100 mg/mL stock solutions of cinnamaldehyde and glutaraldehyde dissolved in dimethyl sulfoxide (DMSO) and ddH_2_O, respectively, were serially diluted in 2 mL of sterile Medium N. 63, to the lowest concentration of 1.56 µg/mL. *D. vulgaris* was grown for 48 h at 37°C in Medium N. 63 and further diluted in each assay tube to achieve a final inoculum of 5 × 10^5^ CFU/mL. The MIC was determined as the lowest amount of antimicrobial agent that did not result in the rise of turbidity and/or in blackish color of the medium caused by FeS precipitation, indicative of bacterial growth (Fig. 1). From each test tube previously used for MIC determination, in which no growth was visible, 1 mL of broth was mixed with 9 mL of molten solid Medium N. 63, poured into a Petri dish and incubated at 37°C for 72 h. The minimum bactericidal concentration (MBC) was determined as the first antibacterial dilution for which no visible growth of *D. vulgaris* as colonies on solid media was obtained at the end of the incubation period. The MBC/MIC ratio was subsequently calculated, considering a MBC/MIC ratio >4 or ≤4, indicative of a bacteriostatic or bactericidal effect, respectively (56).

### *D. vulgaris* biofilm formation and disruption on glass coverslips

*D. vulgaris* biofilms were allowed to form on sterile glass coverslips (13 mm diameter, VWR International s.r.l., Italy) previously treated with 70% v/v ethanol, then washed with ddH_2_O, and air-dried before autoclaving. Briefly, 72 h of the culture of *D. vulgaris* was diluted to an OD_600_ of 0.007 (corresponding to ca. 1 × 10^6^ CFU/mL) in Medium N. 63 and used to inoculate a 24-well flat-bottom microtiter plate equipped with a glass coverslip in each well. Biofilm formation occurred during static incubation at 37°C for 96 h in a BD GasPak EZ Standard Incubation Container to allow *D. vulgaris* growth and was inspected every 24 h by confocal laser scanning microscopy (CLSM). Prior to CLSM visualization, planktonic cells were removed by gently washing each coverslip with sterile PBS for 5 min. Then coverslips were stained in 0.01% w/v acridine orange in PBS for 15 min in the dark and then washed (twice) with PBS for 10 min each. Biofilms were observed with a Leica TCS SP5 confocal microscope equipped with a 40× oil immersion objective, and biofilm spatial characteristics were quantified using COMSTAT version 2.1, analyzing at least five image stacks per condition (57–59).

To investigate the disruptive effect of cinnamaldehyde and glutaraldehyde on *D. vulgaris* biofilms, 72-h-old biofilms were gently washed in PBS to remove planktonic cells and then transferred to a new 24-well flat-bottom microtiter plate containing different cinnamaldehyde and glutaraldehyde dilutions (100, 50, 25, and 12.5 µg/mL) in Medium N. 63. Plates were incubated at 37°C without shaking for further 48 h. Then, CLSM biofilm visualization was performed as described above.

### *D. vulgaris* biofilm formation and disruption on metal coupons

Metal coupons (18 mm diameter, 2 mm of thickness—material code: S355J2G3—chemical composition: 0.20% C, 0.20% Si, 0.037% Al, 0.005% S, 0.02% Cu, 0.98% Mn, 0.009% P, 0.005% N, 0.02% Cr, 0.36% CEV, and 0.01% Ni, Rometec Srl, Roma, Italy) were treated with 70% EtOH for 10 min, washed with ddH_2_O, and dry heat sterilized (3 h at 180°C). Coupons were then placed in a 12-well flat-bottom microtiter plate containing 3 mL of Medium N. 63 inoculated with 1 × 10^6^ CFU/mL of *D. vulgaris*. Biofilm formation was allowed during static incubation at 37°C for 72 h in a BD GasPak EZ Standard Incubation Container to allow *D. vulgaris* growth. For every 24 h, three coupons were removed, washed twice with sterile PBS to remove planktonic cells, and subjected to two rounds of sonication followed by serial dilution and viable count to quantify adherent bacterial cells (Fig. S1).

To investigate the disruptive effect of cinnamaldehyde on *D. vulgaris* biofilms formed on metal coupons, 48-h-old biofilms were gently washed in PBS to remove planktonic cells and then transferred to a new 12-well flat-bottom microtiter plate containing 100 µg/mL of cinnamaldehyde (i.e., 8× MIC) in Medium N. 63. Plates were incubated at 37°C without shaking for further 48 h. Adherent cells were then quantified by viable cell count (CFU/mL), as detailed (Fig. S1).

### Statistical analysis

Statistical analysis was performed with the GraphPad Prism software, version 10.2.0, using one-way ANOVA, followed by Dunn’s multiple-comparison test. Differences among treatments and non-treated controls (NT) were considered statistically significant with a *P*-value ≤ 0.001, indicated with *.

## RESULTS

### Susceptibility of *D. vulgaris* to cinnamaldehyde and glutaraldehyde

*D. vulgaris* susceptibility to cinnamaldehyde and glutaraldehyde was initially investigated by determining the MIC in Medium N. 63. After 72 h of incubation in anoxic conditions at 37°C, bacterial growth was monitored by turbidimetry. Test tubes containing a compound concentration ≤6.125 µg/mL showed a turbidity increase comparable to the one of the positive control devoid of any compound, whereas at concentrations ≥ 12.5 µg/mL, no change in turbidity was recorded in comparison to the negative control (i.e., not-inoculated medium), indicative of bacterial growth inhibition. Thus, the MIC of both cinnamaldehyde and glutaraldehyde was determined to be 12.5 µg/mL (Fig. 1). MBC determination showed lack of bacterial growth at the MIC, leading to a MBC/MIC ratio of 1, indicating a bactericidal activity of both cinnamaldehyde and glutaraldehyde.

**Fig 2:**
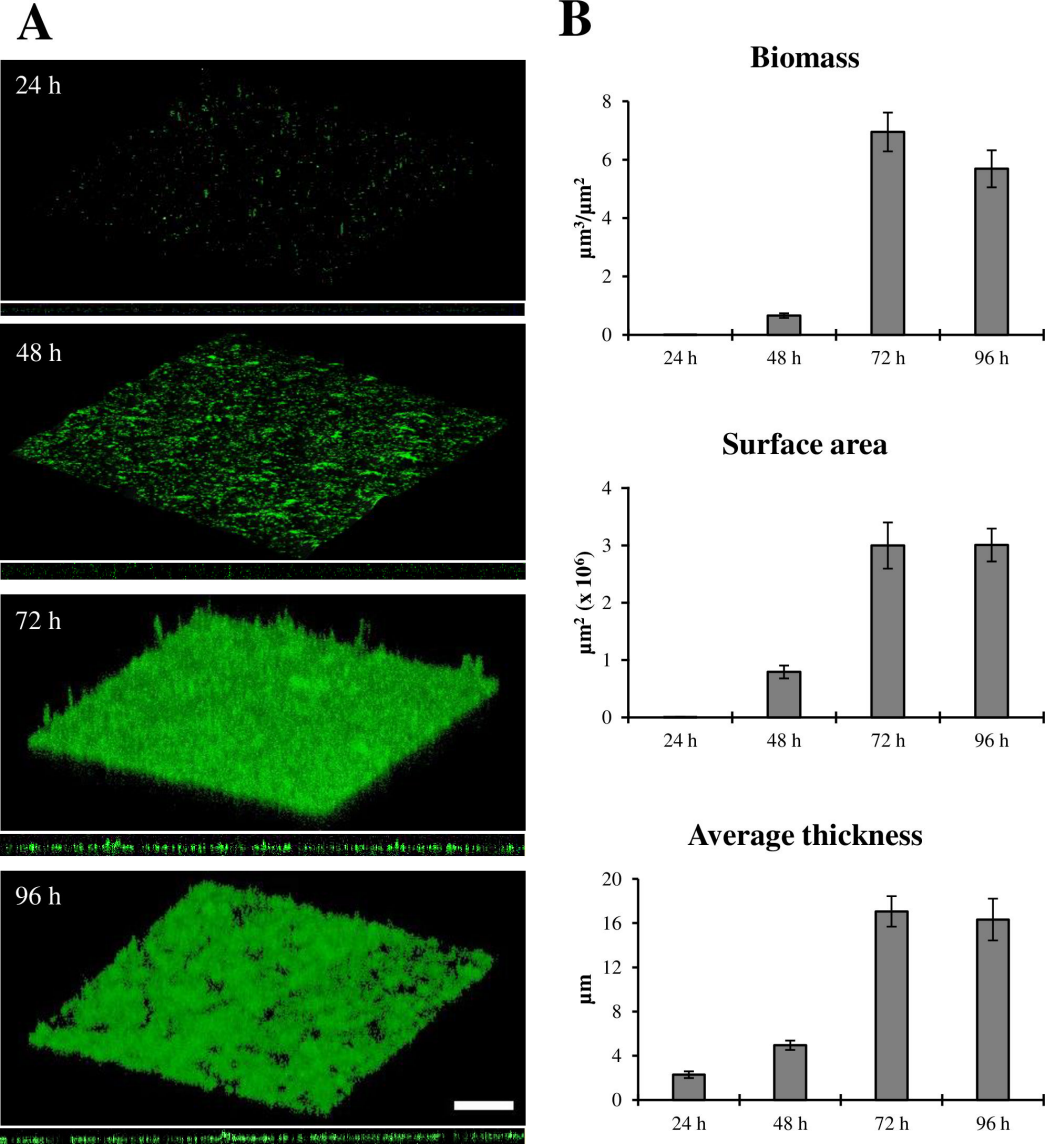
Time course of *D. vulgaris* biofilm formation on glass coverslips. (**A**) Representative confocal microscope images of biofilms (x-y plane and orthogonal view) stained with acridine orange. Scale bar, 50 µm. (**B**) Quantification of biofilm spatial characteristics determined by analysis with COMSTAT, version 2.1. At least five image stacks were analyzed per condition.

To examine the disruptive effect of cinnamaldehyde and glutaraldehyde on *D. vulgaris* biofilm, the biofilm formation dynamics of *D. vulgaris* was carefully investigated over 96 h post-inoculum in Medium N. 63 at 37°C, on glass coverslips using CLSM. Patches of *D. vulgaris* cell aggregates began to appear at 48 h, progressively expanding to obtain a thick and confluent layer after 72 h, followed by the onset of dispersion at 96 h (Fig. 2A). Analyses of biofilm architecture showed that biomass increased progressively up to 72 h, reaching a maximum of ca. 7 µm^3^/µm^2^, along with an increase in thickness and surface area (17 µm and 3 × 10^6^ µm^2^, respectively) (Fig. 2B). The decrease in all biofilm spatial characteristics observed after 96 h is likely due to nutrient exhaustion and catabolite buildup, leading to the dispersal of the mature biofilm, as confirmed by CLSM observations (Fig. 2).

### Effect of cinnamaldehyde and glutaraldehyde against preformed *D. vulgaris* biofilms

**Fig 3:**
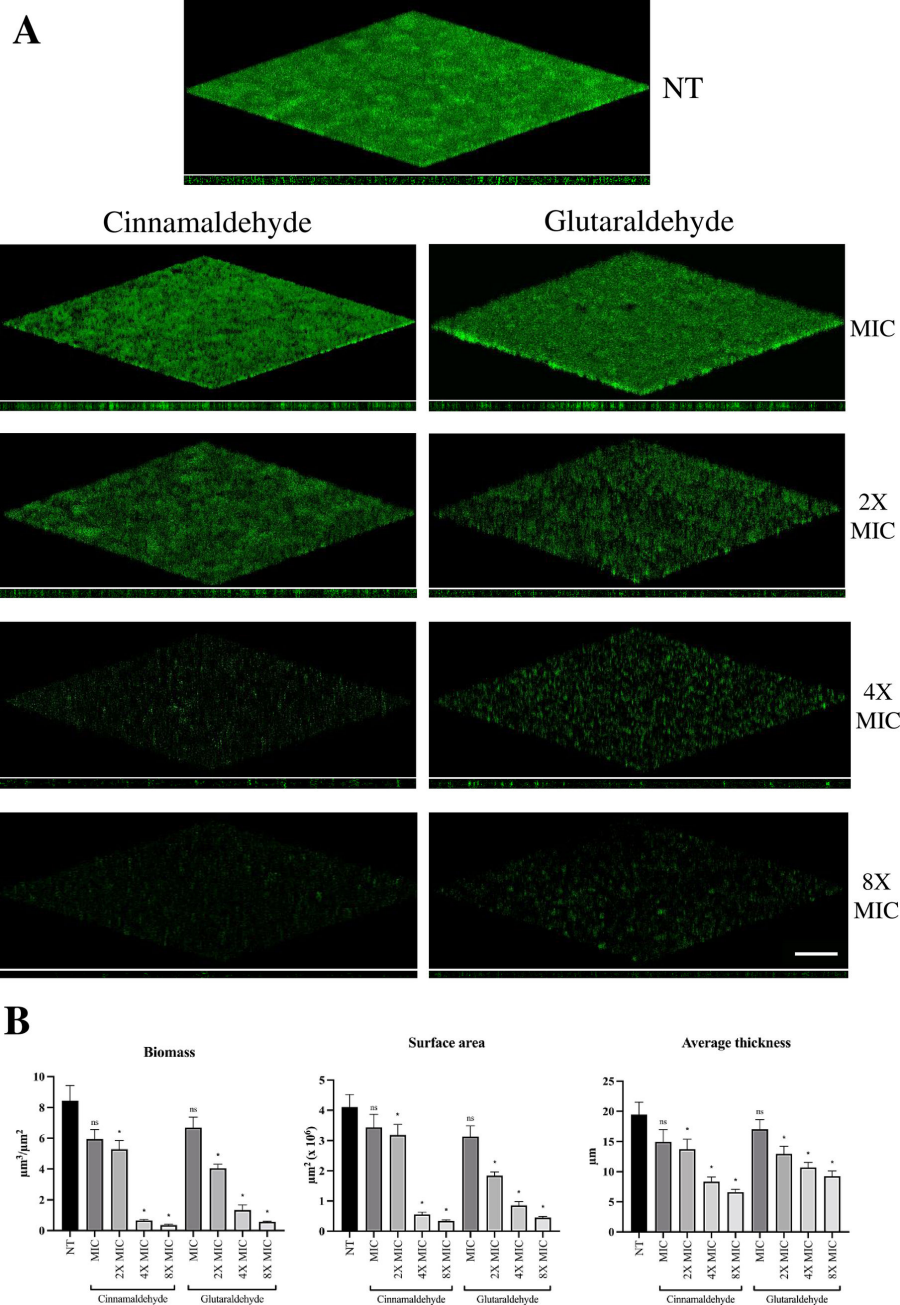
Effect of cinnamaldehyde and glutaraldehyde on 72-h-old *D. vulgaris* biofilms. (**A**) Representative confocal microscope images of *D. vulgaris* biofilm developed for 72 h (top) and treated with cinnamaldehyde (left) and glutaraldehyde (right) concentrations ranging from MIC to 8× MIC (12.5, 25, 50, and 100 µg/mL) for further 48 h at 37°C in anoxic conditions (x-y plane and orthogonal view). Scale bar, 50 µm. (**B**) Quantification of biofilm spatial characteristics determined by analysis with COMSTAT, version 2.1. At least five image stacks were analyzed per condition. Statistical analysis was performed with the GraphPad Prism software, using one-way ANOVA, followed by the Dunn’s multiple-comparison test. Non-treated (NT) controls refer to 72-h-old *D. vulgaris* biofilms exposed for further 48 h in unamended Medium N. 63. Differences among treatments and NT controls were considered highly statistically significant (*) with a *P*-value ≤ 0.001; not significant (ns) indicates *P* > 0.001. Black columns indicate NT controls, and all other columns represent treatments with either cinnamaldehyde or glutaraldehyde, at the indicated concentrations (MIC, 2× MIC, 4× MIC, and 8× MIC).

To investigate the disruptive effect of cinnamaldehyde and glutaraldehyde on *D. vulgaris* preformed biofilms, 72-h-old mature biofilms were exposed to different concentrations of each compound (ranging from the MIC to 8× MIC) for further 48 h, under anoxic conditions, prior to confocal microscopy analysis. Both cinnamaldehyde and glutaraldehyde caused evident disruption of preformed *D. vulgaris* biofilms in a concentration-dependent manner (Fig. 3A), consistent with a reduction in biofilm biomass, surface area, and average thickness (Fig. 3B). Notably, treatment with 4× MIC of cinnamaldehyde and glutaraldehyde caused significant disruption of preformed biofilms compared to the untreated biofilm, with a reduction of ca. 90% vs 85% for biomass, 85% vs 80% for surface area, and 60% vs 45% for average thickness, respectively (Fig. 3B).

**Fig 4:**
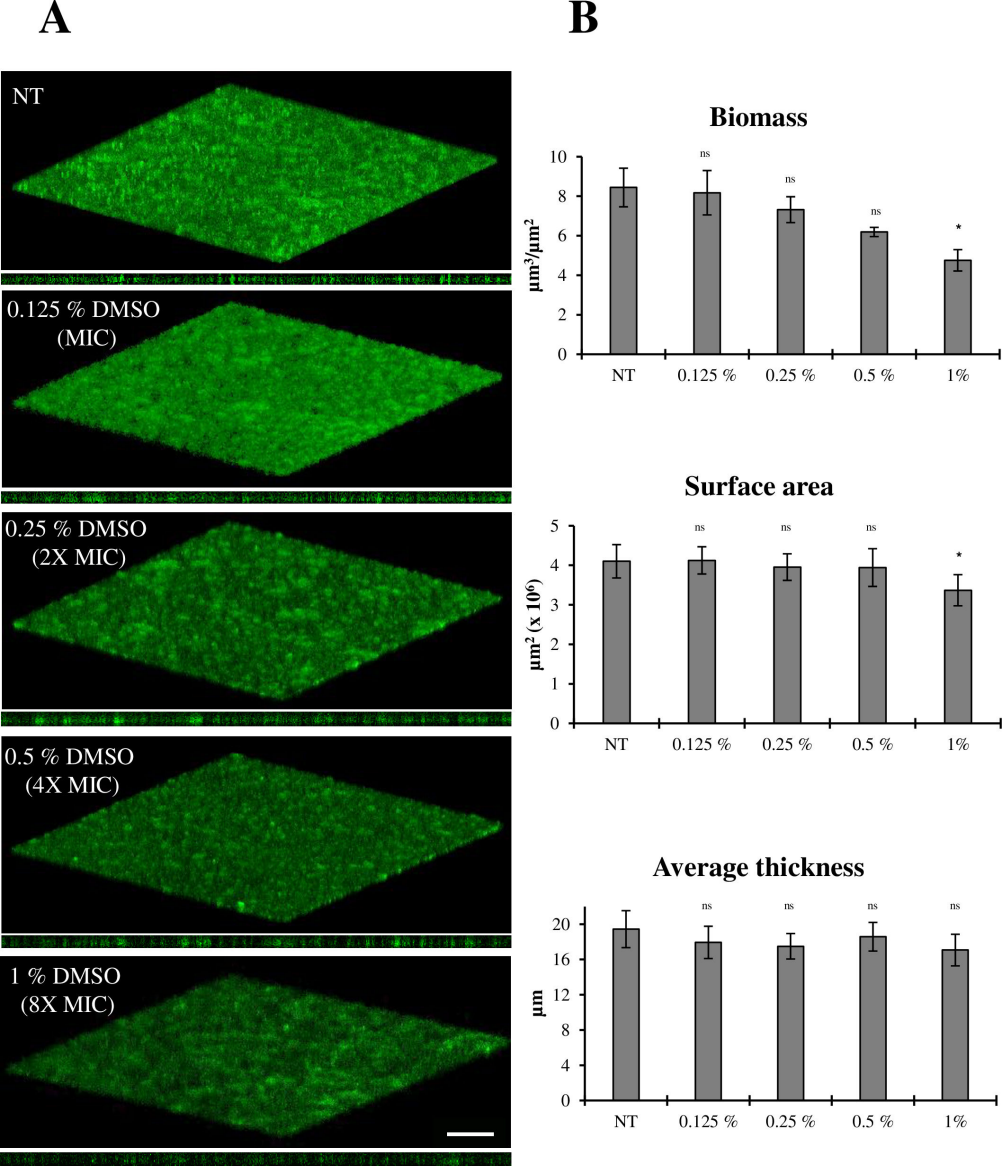
Effect of DMSO on 72-h-old *D. vulgaris* biofilms. (**A**) Representative confocal microscope images of *D. vulgaris* biofilm developed for 72 h and treated or not (NT) for 48 h with DMSO at the same concentrations present during treatments with cinnamaldehyde (0.125%, 0.25%, 0.5%, and 1% of DMSO, corresponding to the amount present at MIC, 2× MIC, 4× MIC, and 8× MIC, respectively). Treatments were performed in anoxic conditions, and biofilms were stained with acridine orange. Scale bar, 50 µm. (**B**) Quantification of biofilm spatial characteristics determined by analysis with COMSTAT, version 2.1. At least five image stacks were analyzed per condition. Statistical analysis was performed with the GraphPad Prism software, using one-way ANOVA, followed by the Dunn’s multiple-comparison test. Non-treated (NT) controls refer to 72 h-old *D. vulgaris* biofilms exposed for further 48 h in unamended Medium N. 63. Differences among treatments and NT controls were considered highly statistically significant (*) with a *P*-value ≤ 0.001; not significant (ns) indicates *P* > 0.001.

Since cinnamaldehyde was dissolved in DMSO, differently from glutaraldehyde which was soluble in ddH_2_O, the potential disruptive effect of DMSO on *D. vulgaris* biofilm was also investigated, to rule out a solvent-dependent effect. To this aim, mature *D. vulgaris* biofilms were exposed to different DMSO concentrations (1%, 0.5%, 0.25%, and 0.125% of DMSO corresponding to the DMSO contained in the 8× MIC, 4× MIC, 2× MIC, and MIC treatments, respectively) for 48 h, under anoxic conditions (Fig. 4A). DMSO at concentrations of 0.125%, 0.25%, and 0.5% did not significantly reduce the biofilm biomass compared to the untreated control, thus confirming the potential of cinnamaldehyde, rather than its solvent, in eradicating pre-formed *D. vulgaris* biofilms. When used at the concentration of 1%, however, DMSO caused a statistically significant reduction in biomass and surface area (ca. 40% and 15% compared to NT, respectively) (Fig. 4B), confirming that DMSO can be toxic to microorganisms and should not be used at concentrations higher than 2% (60).

### *D. vulgaris* biofilm formation and disruption of preformed biofilms on metal coupons

Since SRB-mediated corrosion mainly affects carbon steel surfaces (61), the antibiofilm activity of cinnamaldehyde was investigated on representative metal coupons on which *D. vulgaris* biofilms were allowed to form for 72 h. To this aim, metal coupons inoculated with *D. vulgaris* were initially analyzed for cell adhesion/biofilm formation every 24 h for 72 h. Unlike glass coverslips, biofilm formation on metal coupons could not be analyzed using CLSM due to the weight and thickness of the coupons. Consequently, every 24 h, three inoculated metal coupons were sacrificed, and the biofilm present on each coupon was detached using cycles of vortex and sonication (Fig. S1), and the resulting supernatant was serially diluted for *D. vulgaris* CFU enumeration.

**Fig 5:**
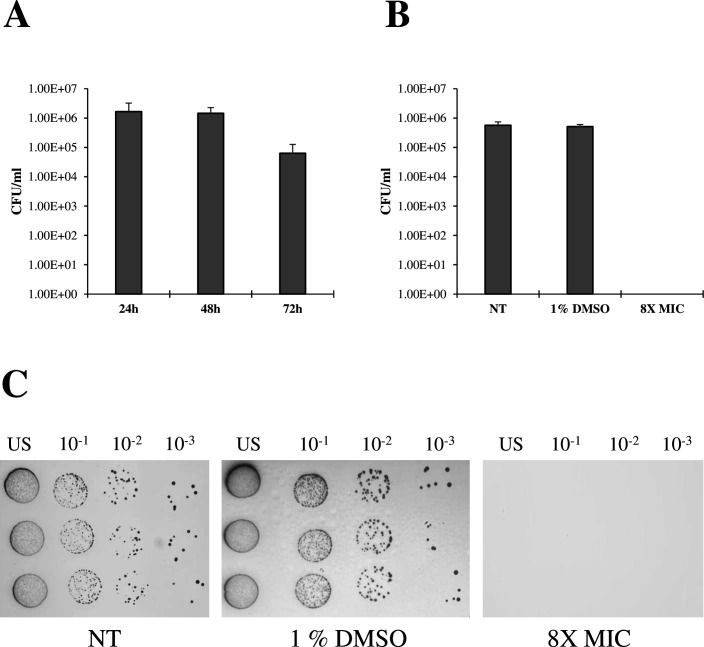
*D. vulgaris* biofilms on metal coupons: formation, disruption, and viable count. (**A**) *D. vulgaris* biofilm formation on metal coupons for 72 h followed by viable count after biofilm detachment. (**B**) Disruption (measured as CFU/mL after biofilm detachment from the metal coupons) of 48-h-old *D. vulgaris* biofilms treated with the maximum concentration of cinnamaldehyde (8× MIC) as well as 1% DMSO, compared to the non-treated (NT) control. (**C**) Representative images of serially diluted (up to 10^−3^, US: undiluted sample) bacterial suspensions (10 µL) obtained after biofilm detachment from metal coupons, spotted onto agar Medium N. 63. NT controls refer to 48-h-old *D. vulgaris* biofilms exposed for further 48 h in unamended Medium N. 63.

After 48 h, *D. vulgaris* viable cell count reached ca. 1.5 × 10^6^ CFU/mL, which decreased to ca. 1 × 10^5^ CFU/mL at 72 h (Fig. 5A). Therefore, the *D. vulgaris* 48-h-old biofilm was chosen as the optimal condition to evaluate the disruptive effect of cinnamaldehyde at the highest concentration tested on cover glasses (i.e., 8× MIC). The same volume of DMSO present at 8× MIC (i.e., 1%) was included as a control, to confirm that the observed effect was solely due to cinnamaldehyde rather than the solvent. *D. vulgaris* 48-h-old biofilms were incubated for an additional 48 h, after which a viable count was performed. Interestingly, no difference was observed between the non-treated (NT) samples and those treated with 1% DMSO (Fig. 5B). By contrast, no CFU/mL were detected at 8× MIC, indicating a bactericidal effect of cinnamaldehyde even on *D. vulgaris* biofilms developed on metal surfaces (Fig. 5C).

## DISCUSSION

Investigating the dynamics of biofilm formation in SRB responsible for microbiologically influenced corrosion is essential for the development of eco-friendly strategies to replace the use of toxic biocides, as part of corrosion mitigation strategies. In this study, we demonstrate for the first time that cinnamaldehyde exhibits promising antibacterial and antibiofilm properties against *D. vulgaris*, an SRB model microorganism. Indeed, cinnamaldehyde showed comparable efficacy to the well-known reference biocide, glutaraldehyde, which is however toxic to the environment, particularly to aquatic ecosystems (29, 62). Cinnamaldehyde is well known for its wide spectrum of antimicrobial activity; however, planktonic growth inhibition of relevant pathogenic species has been documented for concentrations ≥250 µg/mL, including *Escherichia coli* (MIC = 250 µg/mL [63]; MIC = 310 µg/mL [64, 65]; MIC = 780 µg/mL [66]); *Staphylococcus* aureus (MIC = 250 µg/mL [63]; MIC = 310 µg/mL [64]; MIC = 500 µg/mL [67, 68]); *Pseudomonas aeruginosa* (MIC = 250 µg/mL [63]; MIC = 800 µg/mL [69]; MIC = 1,000 µg/mL [68, 70]; MIC = 1,024 µg/mL [71]), and *Enterococcus faecalis* (MIC = 250 µg/mL [67]; MIC = 1,000 µg/mL [68]).

In this work, we found that a >20 times lower concentration of cinnamaldehyde (i.e.*,* 12.5 µg/ml) inhibited the growth and killed planktonic *D. vulgaris* cells (Fig. 1) and, more importantly, a concentration as low as 50 µg/mL (i.e., 4× MIC) almost completely disrupted biofilm-grown cells, by reducing biomass (>90%), surface area (>85%), and thickness (>60%) (Fig. 3). These findings are particularly relevant for strategies aimed at mitigating microbial corrosion since sessile cells within biofilms are notoriously more difficult to eradicate than planktonic ones. Indeed, in field applications, very high concentrations of biocides are typically required to eradicate sessile cells due to biofilms’ multiple defense mechanisms such as the presence of a diffusional barrier that slows biocide penetration, the reduced metabolic rates that limit biocide uptake, and the upregulation of efflux pumps to expel biocides (72).

Since SRB-mediated corrosion mainly affects steel surfaces, the effect of cinnamaldehyde was also tested against *D. vulgaris* 48-h-old biofilms grown on metal coupons. Cinnamaldehyde exposure (100 µg/mL) for 48 h was bactericidal against *D. vulgaris* adherent cells, as revealed by viable count determination (Fig. 5). Differently from cover glass slips (Fig. 4), no difference was observed between untreated samples and those treated with 1% DMSO (Fig. 5). Although the different experimental setting between glass coverslips and metal coupons does not allow a direct comparison on the biofilm-disruptive effect of cinnamaldehyde, the results observed might suggest that while 1% DMSO seems to affect *D. vulgaris* biofilms in terms of spatial features (i.e., biomass and surface area), it does not affect cell viability, as also observed in the preliminary MIC determination experiments (data not shown).

Interestingly, while numerous studies have highlighted the broad-spectrum antibacterial properties of plant extracts and EOs, very few are focused on bacteria directly involved in microbial corrosion, such as SRB. It has been demonstrated that lemongrass essential oil (LEO) and its principal component, citral, can effectively control both planktonic and sessile cultures of *Desulfovibrio alaskensis* strain NCIMB 13491, with a MIC of 170 µg/mL, a >10 times higher concentration than the cinnamaldehyde one used in this study. In addition, both LEO and citral exhibited anti-biocorrosion effects on carbon steel (52). Other major components of EOs, such as linalool, geraniol, nerol, eugenol, R-limonene, and S-limonene, have also been tested against *D. alaskensis* NCIMB 13491, showing MIC values ranging from 78 to 2,500 µg/mL (54), much higher (i.e., from 6 to 200 times) than the cinnamaldehyde concentration effective against *D. vulgaris*.

Given the limited knowledge about the application of EOs or their components on SRB biofilms, the present study aimed to investigate the potential effect of cinnamaldehyde, which has not previously been applied in this specific area of research. The rationale for choosing cinnamaldehyde lies in its well-documented efficacy as a green corrosion inhibitor for steels in acidic environments (34, 73). Indeed, cinnamaldehyde can adsorb onto the metal surface, forming a protective macroscopic film (34, 44–48, 50, 51). This adsorbed layer acts as a barrier, effectively slowing down the corrosive attack from the surrounding aggressive environment (74). Moreover, cinnamaldehyde has also been regarded as a quorum-sensing inhibitor in biofouling seawater bacteria, able to reduce biofilm formation at the high concentration of 1,200 µg/mL (75), and as a potential antifouling coating in combination with the conventionally-used non-toxic polydimethylsiloxane (76).

We have demonstrated that cinnamaldehyde effectively disrupts pre-formed *D. vulgaris* biofilms both on glass coverslips and metal coupons, paving the way for further studies on its long-term effects and performance under different environmental conditions. Cinnamaldehyde’s dual function as both an antimicrobial agent and a corrosion inhibitor makes it an appealing candidate for industrial applications, particularly in environments where SRB-induced corrosion is prevalent. By mitigating both microbial proliferation and metal degradation caused by the corrosive agents produced by SRB metabolism (e.g., H_2_S), cinnamaldehyde may offer a safer and environmentally friendly approach for controlling SRB activity and managing biofilm-related corrosion, making it a viable replacement for more hazardous traditionally used biocides.

## Data Availability

The authors confirm that all data generated or analyzed during this study are included in the paper and its supplemental material or will be made available from the corresponding author upon request.

## SUPPLEMENTAL MATERIAL

Fig. S1 aem.02200-24-s0001.tif Workflow of *Desulfovibrio vulgaris* biofilm formation and disruption on metal coupons.

ASM does not own the copyrights to Supplemental Material that may be linked to, or accessed through, an article. The authors have granted ASM a non-exclusive, world-wide license to publish the Supplemental Material files. Please contact the corresponding author directly for reuse.
