# Supplementary figures and images for: Cinnamaldehyde effectively disrupts Desulfovibrio vulgaris biofilms: potential implication to mitigate microbiologically influenced corrosion
Source: Appl Environ Microbiol. 2025 Apr 28;91(5):e02200-24. doi: 10.1128/aem.02200-24 (PMC12093973; doi:10.1128/aem.02200-24)

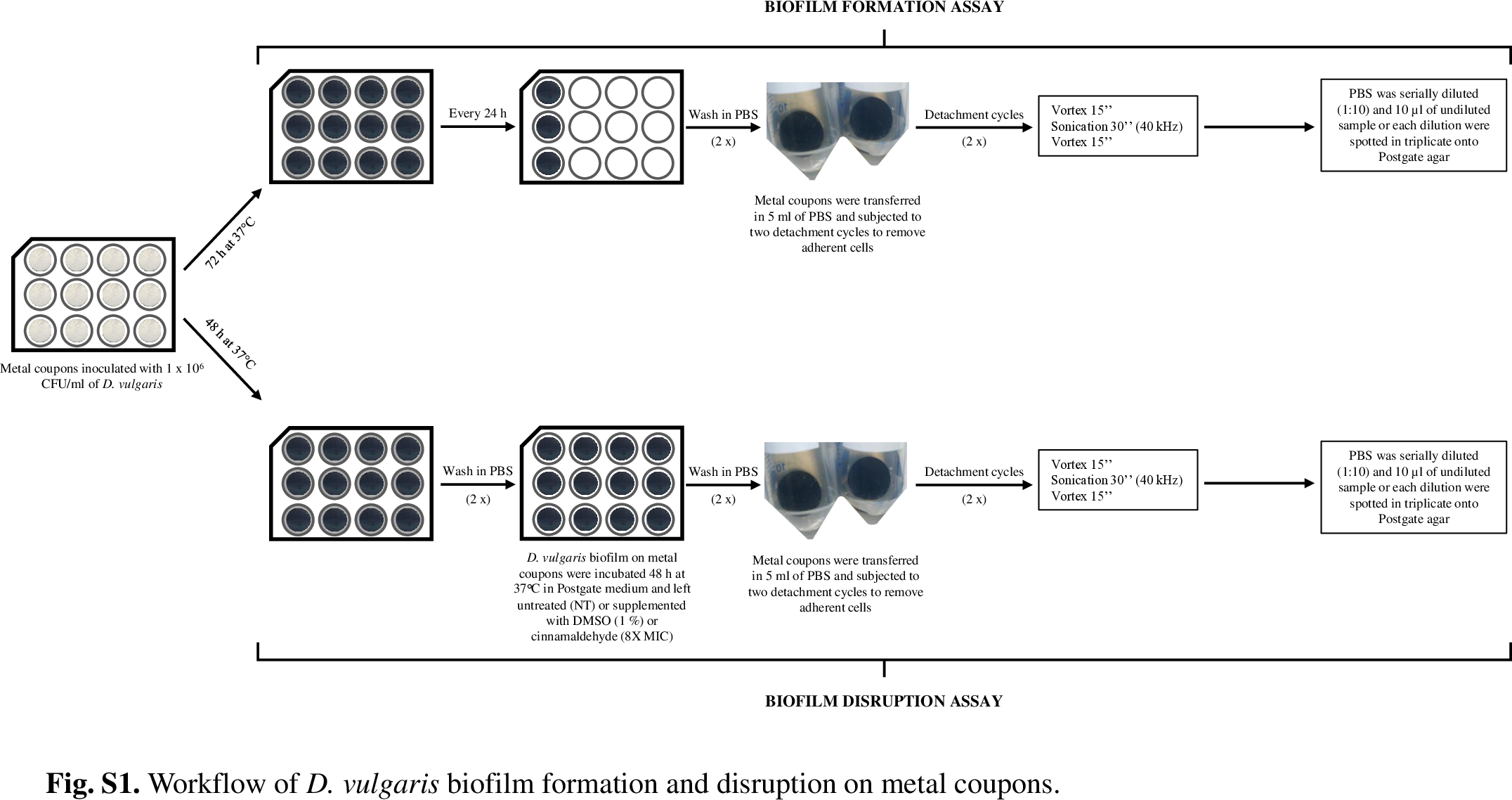

Supplement: Fig. S1 — Workflow of Desulfovibrio vulgaris biofilm formation and disruption on metal coupons. [file aem.02200-24-s0001.tif]
